# Identification of DYNLT1 associated with proliferation, relapse, and metastasis in breast cancer

**DOI:** 10.3389/fmed.2023.1167676

**Published:** 2023-04-04

**Authors:** Sen Miao, Gaoda Ju, Chonghua Jiang, Bing Xue, Lihua Zhao, Rui Zhang, Han Diao, Xingzhou Yu, Linlin Zhang, Xiaozao Pan, Hua Zhang, Lijuan Zang, Lei Wang, Tianhao Zhou

**Affiliations:** ^1^Department of Pathology, Affiliated Hospital of Jining Medical University, Jining, China; ^2^Department of Medical Oncology, Key Laboratory of Carcinogenesis and Translational Research (Ministry of Education/Beijing), Peking University Cancer Hospital and Institute, Beijing, China; ^3^Department of Neurosurgery, Affiliated Haikou Hospital of Xiangya Medical College, Central South University, Haikou, China; ^4^Department of Pathology Center, Shanghai General Hospital, Shanghai Jiaotong University School of Medicine, Shanghai, China; ^5^Department of Breast Surgery, Affiliated Hospital of Jining Medical University, Jining, China; ^6^Department of Medical Oncology, Shanghai First People's Hospital, Shanghai Jiao Tong University School of Medicine, Shanghai, China

**Keywords:** DYNLT1, breast cancer, biomarker, prognosis, immune checkpoint blocking therapy

## Abstract

**Background:**

Breast cancer (BC) is the most common malignant disease worldwide. Although the survival rate is improved in recent years, the prognosis is still bleak once recurrence and metastasis occur. It is vital to investigate more efficient biomarkers for predicting the metastasis and relapse of BC. DYNLT1 has been reported that participating in the progression of multiple cancers. However, there is still a lack of study about the correlation between DYNLT1 and BC.

**Methods:**

In this study, we evaluated and validated the expression pattern and prognostic implication of DYNLT1 in BC with multiple public cohorts and BC tumor microarrays (TMAs) of paraffin-embedded tissues collected from the Affiliated Hospital of Jining Medical University. The response biomarkers for immune therapy, such as tumor mutational burden (TMB), between different DYNLT1 expression level BC samples were investigated using data from the TCGA-BRCA cohort utilizing public online tools. In addition, colony formation and transwell assay were conducted to verify the effects of DYNLT1 in BC cell line proliferation and invasion.

**Results:**

The results demonstrated that DYNLT1 overexpressed in BC and predicted poor relapse-free survival in our own BC TMA cohort. In addition, DYNLT1 induced BC development by promoting MDA-MB-231 cell proliferation migration, and metastasis.

**Conclusion:**

Altogether, our findings proposed that DYNLT1 could be a diagnostic and prognostic indicator in BC.

## 1. Introduction

Breast carcinoma (BC) is the most common cancer in women and ranks the second leading cause of tumor-related death in women in the United States and in China (1, 2). According to the molecular pathological types, there are various therapeutic strategies for BC, such as surgical therapy, radiation therapy, chemotherapy, endocrine therapy, and targeted therapy (3). The response to treatment and prognosis of BC relies on molecular characteristics that have been well established, and the molecular type based on estrogen receptor (ER), progesterone receptor (PR), and human epidermal growth factor receptor-2 (HER-2) status shows excellent performance for guiding clinicians to select the optimal treatment for BC patients in the past few decades (4, 5). In addition, with the development of modern genomic and transcriptomic technologies, numerous gene markers are identified for predicting the response to treatment and prognosis of cancer (6, 7). The prognosis of BC is improved in the past few decades; however, there are still numerous women dying from BC, especially triple-negative breast cancer (TNBC), in the world. As a result, it is urgent to explore a more specific molecular target to direct the diagnosis and treatment of BC.

In this study, we identified that dynein light chain tctex type 1 (DYNLT1), a component of the cytoplasmic dynein 1 complex, may predict the prognosis of BC. DYNLT1 is responsible for the intracellular retrograde motility of vesicles and organelles along microtubules, binding to transport cargo, and is involved in apical cargo transport. It is reported that DYNLT1 plays an important role in many biological functions and diseases, such as Huntington's disease (8), fertilizing potential of human spermatozoa (9), migration of epidermal cells in hypoxia (10), autophagy lysosomal degradation (11), and several types of cancer. DYNLT1 has been reported to promote glioblastoma progression and is associated with tumor-node-metastasis (TNM) grade (12). In gastric cancer (GC), DYNLT1 takes part in the miR-15b-3p/Caspase-3/Caspase-9 signaling pathway to promote malignant transformation (13). However, it is still unclear whether DYNLT1 is related to BC.

In this study, we evaluated the mRNA expression of *DYNLT1* between BC and normal breast tissues from multiple public cohorts and validated the results at the protein level by immunohistochemistry (IHC) staining for DYNLT1 in 68 BC samples along with paired 55 adjacent normal breast specimens collected from the Affiliated Hospital of Jining Medical University. In addition, we observed that DYNLT1 affected the migratory and colony-forming abilities of BC cells *in vitro*. Furthermore, *in vivo* experiment was conducted to verify that DYNLT1 knockdown suppressed tumor growth and abolished distant metastasis. Therefore, we proposed that DYNLT1 may have the potential to become a promising diagnostic indicator and prognostic predictor of BC patients.

## 2. Methods and materials

### 2.1. Data acquisition

A total of four Gene Expression Omnibus (GEO) cohorts (GSE15852, GSE9309, GSE109169, and GSE53752) (14–17) were downloaded from the GEO website (https://www.ncbi.nlm.nih.gov/geo) for evaluating the mRNA expression of *DYNLT1* between BC and normal breast tissues.

### 2.2. GEPIA 2.0 database

The GEPIA 2.0 (http://gepia2.cancer-pku.cn/#index) database (18) was utilized to evaluate the mRNA expression of *DYNLT1* in pan cancers using data from The Cancer Genome Atlas (TCGA). In addition, GEPIA 2.0 was also utilized to evaluate the prognostic implication of *DYNLT1* in pan cancers.

### 2.3. Breast cancer gene-expression miner database

The Breast Cancer Gene-Expression Miner (http://bcgenex.centregauducheau.fr/BC-GEM/GEM-Accueil.php?js=1) database (19) was utilized to evaluate the mRNA expression of *DYNLT1* in subgroups of BC samples stratified based on multiple clinic-pathological features.

### 2.4. PrognoScan database

The PrognoScan (http://dna00.bio.kyutech.ac.jp/PrognoScan/index.html) database (20) was utilized to evaluate the prognostic implication of *DYNLT1* in cancer.

### 2.5. Protein–protein interaction analysis

DYNLT1 was inputted into the String (https://www.string-db.org/) database (21), and a PPI network was successfully outputted.

### 2.6. GO and KEGG analyses

DYNLT1 and its potential interacting proteins were utilized to perform gene ontology (GO) and Kyoto Encyclopedia of Genes and Genomes (KEGG) analyses by R software with the “clusterProfiler” package (22). Terms with a false discovery rate (FDR) of <0.05 were illustrated.

### 2.7. CAMOIP database

The CAMOIP (http://camoip.net/) database (23) was utilized to perform GSEA analysis, immune infiltration analysis, and immunogenicity analysis with data from TCGA-BRCA. Tumor mutational burden (TMB), neoantigen load, TGF-beta response score, tumor-infiltrating lymphocytes (TILs) regional fraction, and immune cells' infiltration ratio calculated by CIBERSORT were compared between DYNLT1 high and DYNLT1 low BC samples.

### 2.8. ciBorPortal database

The ciBorPortal (http://www.cbioportal.org/) database (24, 25) was utilized to perform mutational analysis with data from TCGA-BRCA (Firehose Legacy). Core DNA damage repair (DDR)-related genes and their corresponding pathways were extracted from a previous study (26), such as base excision repair (BER), nucleotide excision repair (NER), mismatch repair (MMR), Fanconi anemia (FA), homologous recombination (HR), non-homologous end joining (NHEJ), direct repair (DR), translesion synthesis (TLS), and damage sensor. A sample with pathway mutation means that at least one DDR-related gene in the pathway is mutated.

### 2.9. CancerSEA database

The CancerSEA (http://biocc.hrbmu.edu.cn/CancerSEA/home.jsp) database was utilized to evaluate the correlation of *DYNLT1* expression with 14 functional states of single BC cells (27) using data from the GSE75367 cohort (28).

### 2.10. Human BC specimens

A total of 68 BC samples paired with 55 normal breast tissues collected from the Affiliated Hospital of Jining Medical University were approved by the Ethics Committee of the Affiliated Hospital of Jining Medical University (approval number: 2021-08-C015). All participants provided written informed consent.

### 2.11. Cell culture

MDA-MB-231 and HEK 293T cells were cultured in the DMEM medium (Gibco) with 10% fetal bovine serum (FBS) (Gibco) and 1% penicillin–streptomycin (Gibco) at 37°C with 5% CO_2_.

### 2.12. Western blotting

Cells were lysed by denatured buffer and quantified by the Pierce BCA protein assay (Thermo Scientific). The whole cell lysate protein was separated by SDS-PAGE, transferred to NC membranes (Millipore), blocked by no-fat milk, and then detected by primary antibody DYNLT1 (Proteintech, 11954-1-AP, 1:2000) and HRP-conjugated secondary antibody (Sigma), followed by being exposed to enhanced chemiluminescence (Vazyme). β-actin (Abclonal, AC026, 1:10000) was used as a loading control.

### 2.13. Plasmids and *Lentivirus* production

Annealing and ligation of the DYNLT1-knockdown shRNA were performed and inserted into the enzyme cut pLKO.1. The shDYNLT1 plasmids were then transinfected into HEK293T cells along with psPAX and pMD2.0G. Next, *Lentivirus* was collected to infect MDA-MB-231 cells. The primer sequences are shown as follows: DYNLT1-sh1-F: CCGGGAGGCTATAGAAAGCGCAATTCTCGAGAATTGCGCTTTCTATAGCCTCTTTTTG, DYNLT1-sh1-R: AATTCAAAAAGAGGCTATAGAAAGCGCAATTCTCGAGAATTGCGCTTTCTATAGCCTC; DYNLT1-sh2-F: CCGGCCACAAATGTAGTAGAACAAACTCGAGTTTGTTCTACTACATTTGTGGTTTTTTG, DYNLT1-sh2-R: AATTCAAAAAACCACAAATGTAGTAGAACAAACTCGAGTTTGTTCTACTACATTTGTGG.

### 2.14. IHC assay

Immunohistochemistry (IHC) staining for DYNLT1 (Proteintech, 11954-1-AP, 1:500) was operated by the standard IHC protocol as described earlier (29). The IHC score (values 0–12) was determined by multiplying the score for staining intensity with the score for the frequency of positive staining cells of DYNLT1. Staining intensity was defined as follows: (0) negative; (1) weak; (2) moderate; and (3) strong. The frequency of positive cells was defined as follows: <5%, 0; 5%−25%, 1; 26%−50%, 2; 51%−75%, 3; and more than 75%, 4 (30).

### 2.15. Colony-forming assay

MDA-MB-231 cells were seeded at a density of 200 cells per well in six-well plates. Single cells were cultured in DMEM with 10% FBS at 37°C with 5% CO_2_ for 3 weeks. Colonies were fixed with 10% formalin and then stained with 0.1% crystal violet.

### 2.16. Cell growth assay

Lentivirus-infected stable cells were seeded into 96-well plates and cultured in 10% FBS DMEM (2,000 cells per well, five parallel wells). Then, the cells were collected at different points in time, and the cell number in each well was counted by the CCK-8 reagent. The absorbance at 450 nm was employed to determine the number of viable cells.

### 2.17. Transwell assay

The migration assays and invasion assays were performed using a transwell chamber (Corning). A total of 2 × 10^4^ cells per well were seeded into the upper chamber for the migration assays, while 5 × 10^4^ cells per well were seeded into the upper chamber after matrigel was coagulated at 37°C for the invasion assays with serum-free medium, and the bottom of the chamber contained the DMEM medium with 10% FBS. Cells were fixed by 10% formalin and stained by 0.1% crystal violet after migration for 24 h. Migrated BC cells' pictures were captured by an inverted light microscope at × 100 magnification, and three random fields were counted.

### 2.18. Tumor models

Female SCID mice (6 weeks old) were purchased from the Shanghai Model organism. SCID mice were injected in the right lower breast fat pad with MDA-MB-231 cells knockdown DYNLT1 or vector shRNA control (1 × 10^6^ cells per mouse). Tumor volume was measured every 7 days and calculated according to the formula as follows: volume = 0.5 × tumor length × width × width. Mice were generally sacrificed when tumors became necrotic or their volume reached 1,500 mm^3^, recorded as death for the survival curve. The lung and the liver of dead mice were excised and fixed in formalin. Paraffin-embedded lungs were systematically sectioned and stained with hematoxylin and eosin (H&E) staining, and images were captured by Leica Aperio CS2.

### 2.19. Statistical analysis

Student's *t*-test and chi-square test were utilized to analyze the difference between the two groups. A *P-*value of <0.05 was considered statistically significant.

## 3. Results

### 3.1. DYNLT1 expression was higher in BC compared to normal breast tissues

**Figure 1:**
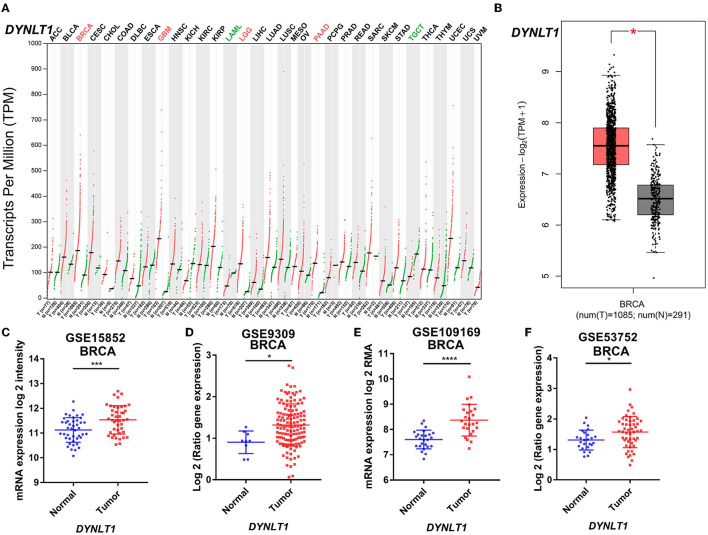
*DYNLT1* mRNA expression was higher in BC tissues compared to normal breast tissues. **(A)** *DYNLT1* mRNA expression across 33 cancer types (TCGA). **(B)** *DYNLT1* mRNA expression between BC tissues and normal breast tissues in the TCGA-BRCA cohort. **(C)** *DYNLT1* mRNA expression between BC tissues and normal breast tissues in the GSE15852 cohort. **(D)** *DYNLT1* mRNA expression between BC tissues and normal breast tissues in the GSE9309 cohort. **(E)** *DYNLT1* mRNA expression between BC tissues and normal breast tissues in the GSE109169 cohort. **(F)** *DYNLT1* mRNA expression between BC tissues and normal breast tissues in the GSE53752 cohort. TCGA, The Cancer Genome Atlas; *P < 0.05, ***P < 0.001, and ****P < 0.0001.

**Figure 2:**
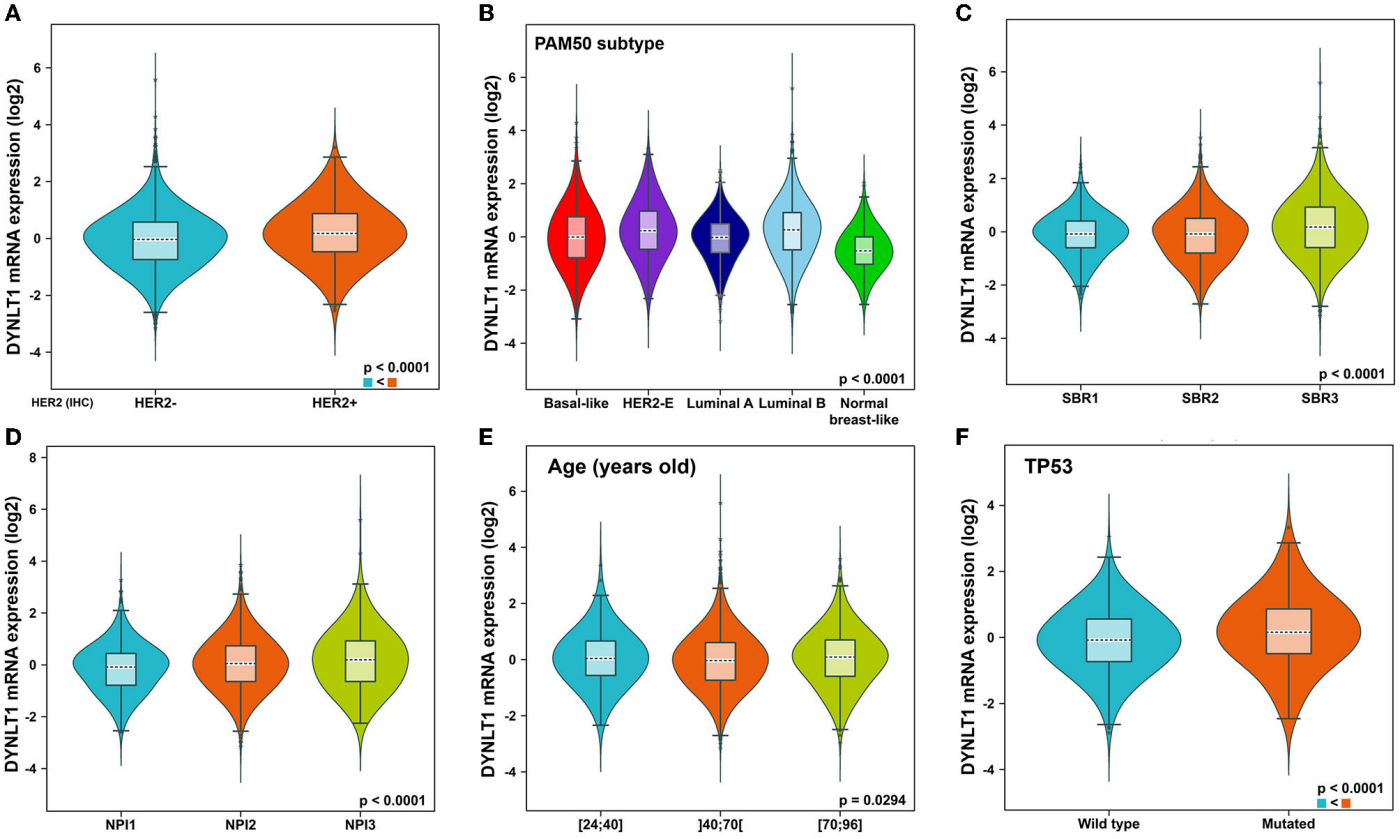
Subtype analysis of DYNLT1 mRNA expression based on clinic-pathological features of BC tissues. **(A)** Subtype analysis of DYNLT1 mRNA expression based on HER2 status. **(B)** Subtype analysis of DYNLT1 mRNA expression based on PAM50 subtype. **(C)** Subtype analysis of DYNLT1 mRNA expression based on SBR grade. **(D)** Subtype analysis of DYNLT1 mRNA expression based on NPI. **(E)** Subtype analysis of DYNLT1 mRNA expression based on age. **(F)** Subtype analysis of DYNLT1 mRNA expression based on TP53 mutant status. HER-2, human epidermal growth factor receptor-2; SBR, Scarff-Bloom-Richardson; NPI, Nottingham prognostic index.

First, we evaluated the mRNA expression of *DYNLT1* across 33 cancer types and paired normal samples with data from TCGA by the GEPIA database. Our results demonstrated that the mRNA expression of *DYNLT1* was higher in most types of cancer tissues compared with paired normal samples, such as BC, GBM, LGG, and PAAD (Figures 1A, B). Next, four GEO cohorts were utilized to validate the result that the mRNA expression of *DYNLT1* was higher in BC tissues compared to paired normal breast samples (Figures 1C–F). In addition, subgroup analysis of multiple clinic pathological features of BC samples in the Breast Cancer Gene-Expression Miner database showed that DYNLT1 expression is related to HER-2 status, PAM50-based intrinsic subtype, Scarff-Bloom-Richardson (SBR) grade, Nottingham prognostic index (NPI), and age and mutation status of TP53 (Figures 2A–F).

### 3.2. High expression of *DYNLT1* predicting poor overall relapse and distant metastasis-free survival of BC

**Figure 3:**
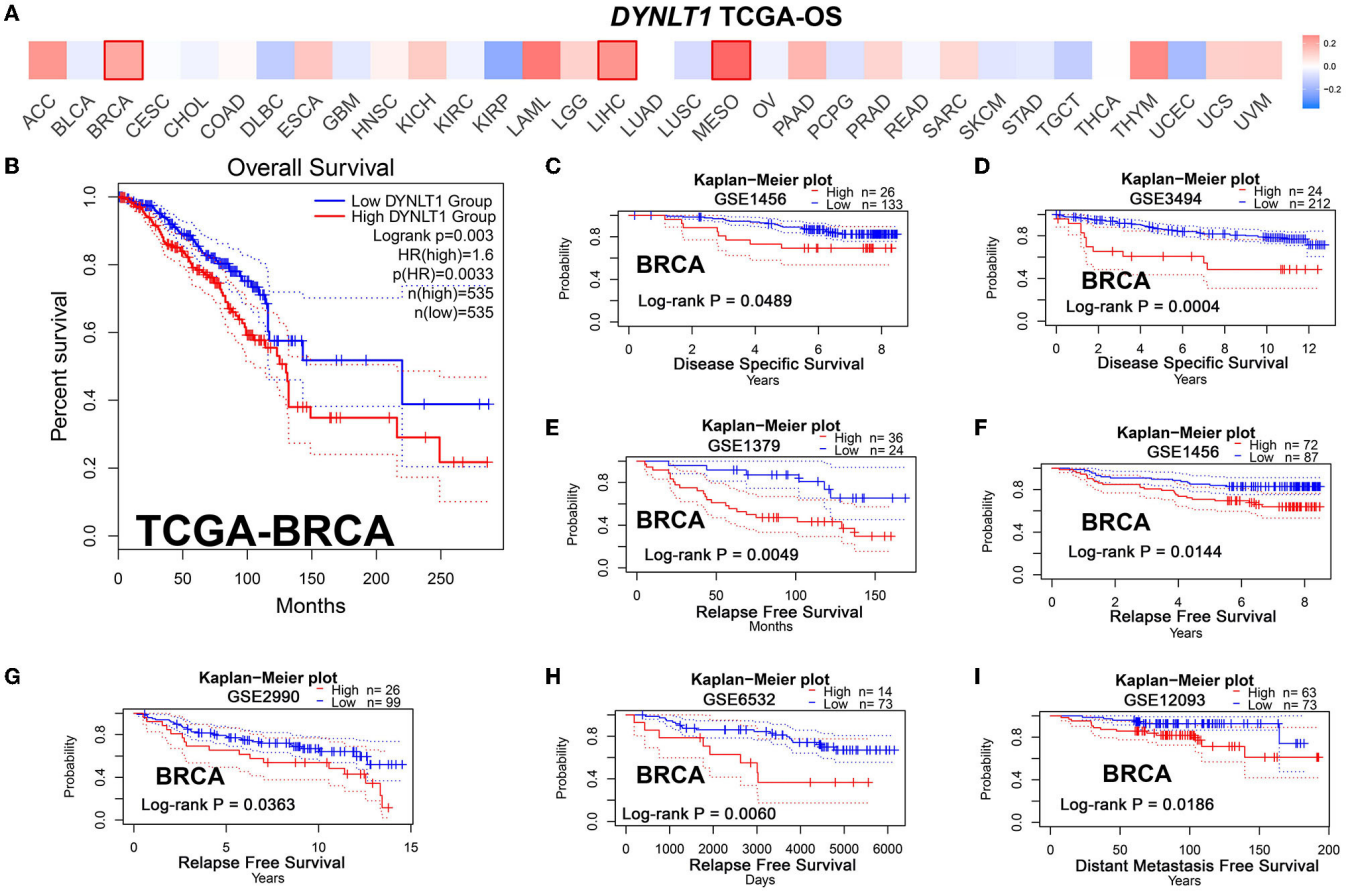
High DYNLT1 expression predicted poor survival in BC. **(A)** High expression predicted poor prognosis in BC, LGG, and LIHC in the GEPIA database. **(B)** High DYNLT1 expression predicted poor overall survival (OS) of BC patients in the TCGA-BRCA cohort. **(C, D)** High DYNLT1 expression predicted poor disease-specific survival (DSS) of BC patients in the GSE1456 cohort **(C)** and GSE3494 cohort **(D)**. **(E–H)** High DYNLT1 expression predicted poor relapse-free survival (RFS) of BC patients in the GSE1379 cohort **(E)**, GSE1456 cohort **(F)**, GSE2990 cohort **(G)**, and GSE6532 cohort **(H)**. **(I)** High DYNLT1 expression predicted poor distant metastasis-free survival (DMFS) of BC patients in the GSE12093 cohort.

First, we evaluated the prognostic implications of *DYNLT1* in 33 cancer types using data from TCGA by the GEPIA database. Our results demonstrated that *DYNLT1* was a significant (*P* < 0.05) risk factor for the prognosis of BC, MESO, and LIHC (Figures 3A, B). Furthermore, we validated the prognostic implication of *DYNLT1* in BC by the PrgnoScan database. Our results identified that high expression of *DYNLT1* predicted poor disease-specific survival (Figures 3C, D), relapse-free survival (Figures 3E–H), and distant metastasis-free survival (Figure 3I) in BC.

### 3.3. Validated the expression pattern and prognostic implication of BC by TMA

**Figure 4:**
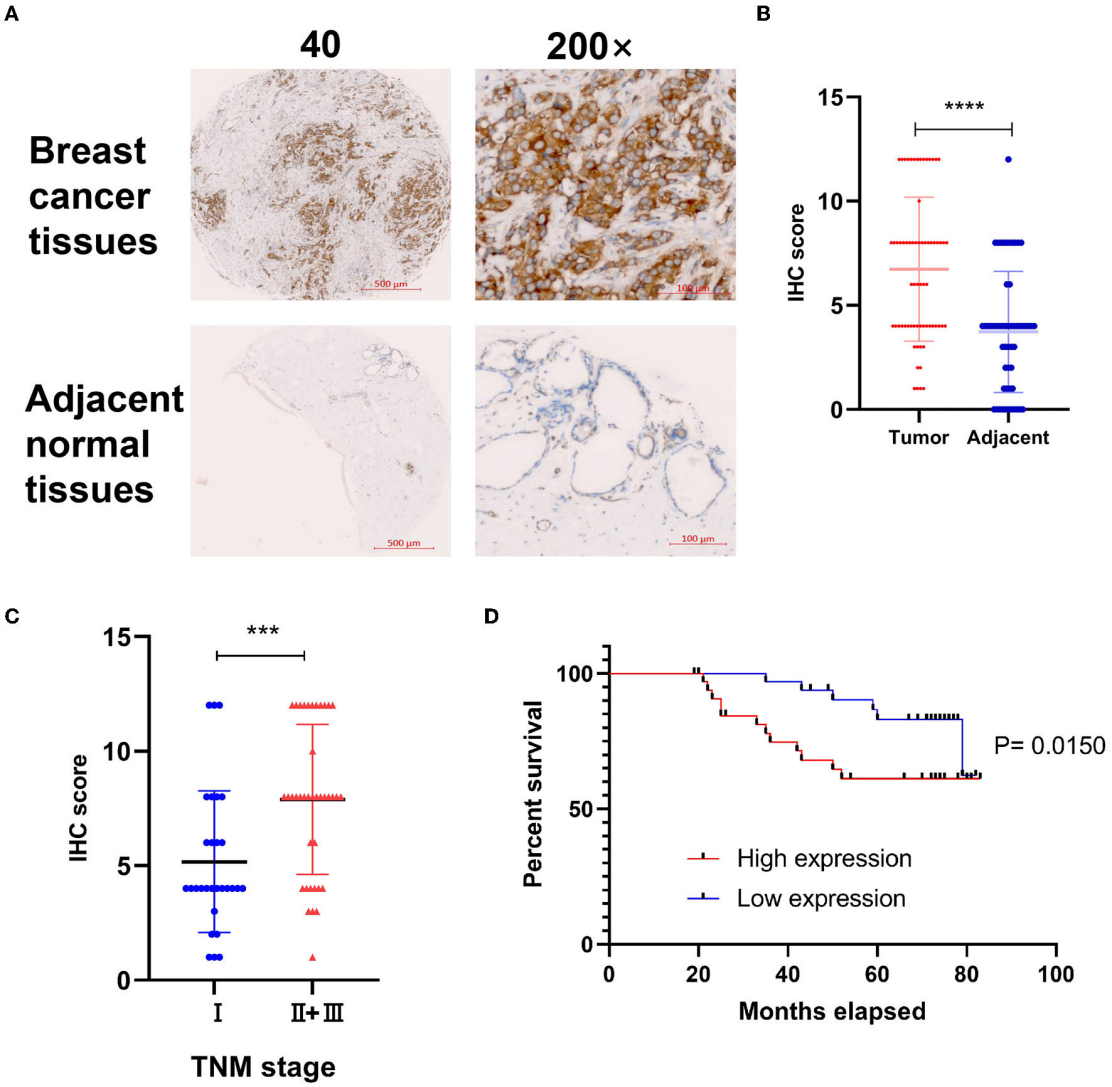
Validated the protein expression pattern and prognostic implication of DYNLT1 in BC. **(A)** Protein expression of DYNLT1 was higher in BC compared to normal breast tissues. **(B)** Scatter plot of TMED IHC score between BC and normal breast tissues. **(C)** Scatter plot of TMED IHC score between TNM stage I BC and TNM stage II+III BC tissues. **(D)** Kaplan–Meier analysis was utilized to compare the relapse-free survival between high DYNLT1 expression BC patients and low DYNLT1 expression BC patients. IHC, immunohistochemistry. ***P < 0.001 and ****P < 0.0001.

To explore the correlation of DYNLT1 and the characteristic of BC patients at the protein level, a BC TMA, containing 68 cancer tissues were used for IHC staining for DYNLT1. The results validated that DYNLT1 expressed significantly higher in BC tissues than in adjacent normal breast samples (Figures 4A, B). High expression of DYNLT1 was positively correlated with higher TNM stage (Figure 4C) and predicted poor relapse-free survival (RFS) with a log-rank *P*-value of <0.015 (Figure 4D).

### 3.4. PPI network and functional annotation of DYNLT1

**Figure 5:**
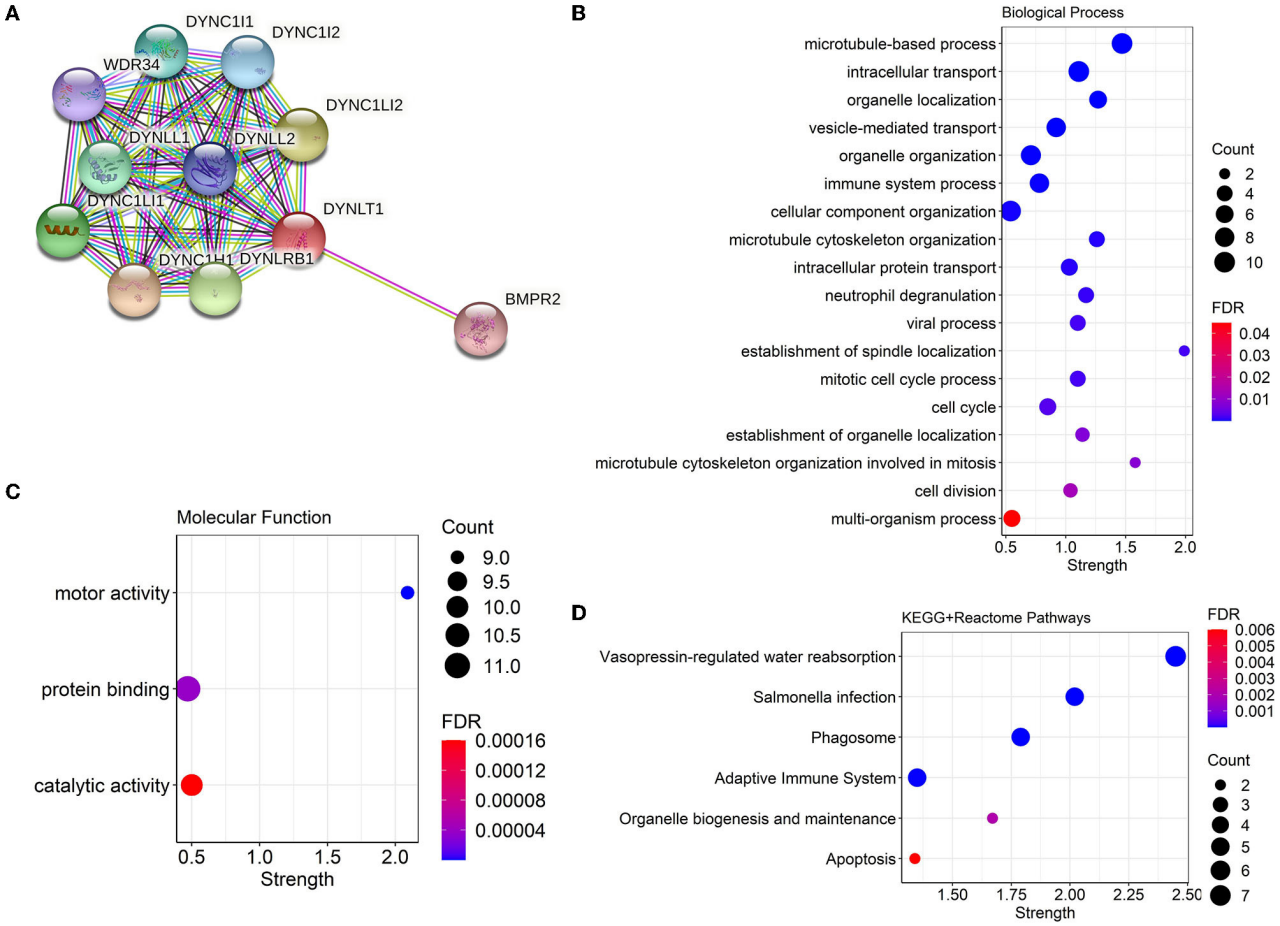
PPI network and functional annotation of DYNLT1. **(A)** PPI network based on DYNLT1. **(B, C)** Gene ontology enrichment analysis: biological process **(B)** and molecular function **(C)**. **(D)** KEGG pathway enrichment analysis. PPI, protein–protein network; GO, gene ontology; KEGG, kyoto encyclopedia of genes and genomes.

We constructed a PPI network combining DYNLT1 and its potential interacting proteins by the String database. The result indicated that DYNLT1 may be capable of interacting with BMPR2, DYNC1H1, DYNC1I1, DYNC1I2, DYNC1LI1, DYNC1LI2, DYNLL1, DYNLL2, DYNLRB1, and WDR34 (Figure 5A). The gene ontology and pathway functional enrichment analyses of genes in this network demonstrated that these genes may involve in cell cycle, cell division, immune system process, vesicle-mediated transport, motor activity, protein binding, phagosome, adaptive immune system, and apoptosis (Figures 5B–D).

### 3.5. DYNLT1 as a predictive biomarker for immune checkpoint blocking therapy in patients with BC

**Figure 6:**
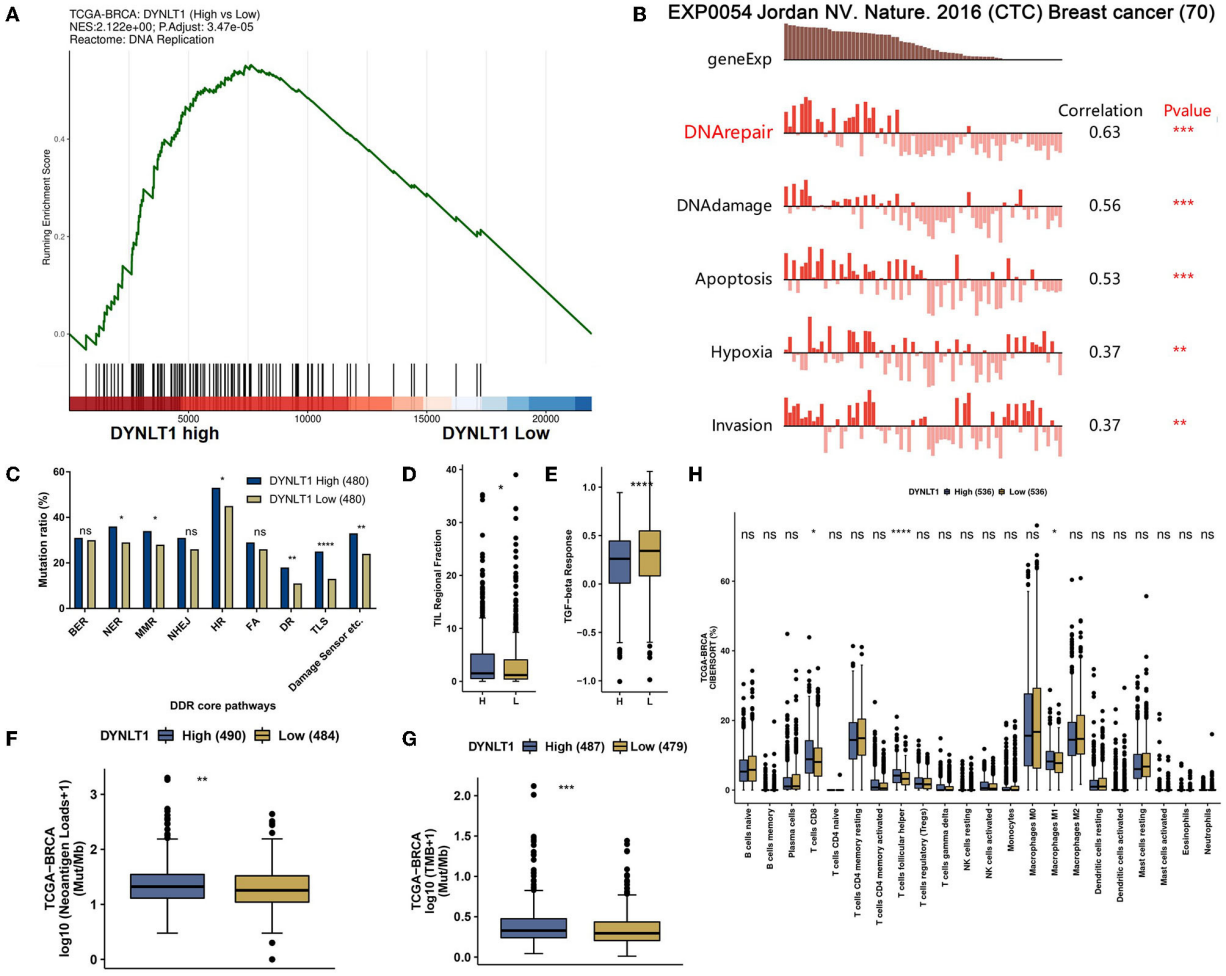
DYNLT1 may predict well overcome in immune checkpoint blocked therapy for patients with BC. **(A)** GSEA analysis showed that the DNA replication pathway was enriched in DYNLT1 High BC samples. **(B)** DTNLT1 mRNA expression was positively related to DNA damage, DNA repair, apoptosis, hypoxia, TILs, and invasion at the single BC cell level. **(C)** The DDR pathways' mutational ratio between DYNLT1 High and DYNLT1 Low BC samples. **(D)** The regional fraction between DYNLT1 High and DYNLT1 Low BC samples. **(E)** The TGF-beta response score between DYNLT1 High and DYNLT1 Low BC samples. **(F)** The neoantigen loads between DYNLT1 High and DYNLT1 Low BC samples. **(G)** The TMB between DYNLT1 High and DYNLT1 Low BC samples. **(H)** The infiltration ratio of immune cells between DYNLT1 High and DYNLT1 Low BC samples. TMB, tumor mutational burden; DDR, DNA damage repair; TILs, tumor-infiltrating lymphocytes; BER, base excision repair; NER, nucleotide excision repair; MMR, mismatch repair; FA, Fanconi anemia; HR, homologous recombination; NHEJ, non-homologous end joining; DR, direct repair; TLS, translesion synthesis; *P < 0.05; **P < 0.01; ***P < 0.001; and ****P < 0.0001.

Generally, a high DNA damage repair (DDR) mutational ratio, a high proportion of TMB, high neoantigen loads, high TILs regional fraction, and low TGF-beta response predict well overcome in ICB therapy for patients with cancer (31, 32). In our study, the results of GSEA analysis based on data from TCGA-BRCA showed that DNA replication (Figure 6A) pathways were enriched in DYNLT1 high BC samples. In addition, we found that DTNLT1 expression was positively related to DNA damage and DNA repair (Pearson's correlation > 0.3, *P* < 0.05) at the single cell level of BC by the CancerSEA database (Figure 6B). We found that BC patients in the DYNLT1 high group had higher levels of DDR mutational ratio (Figure 6C), TILs regional fraction (Figure 6D), and lower TGF-beta response (Figure 6E) compared to those in the DYNLT1 low group. Moreover, a high proportion of neoantigen loads (Figure 6F) and TMB (Figure 6G) was shown in the DYNLT1 high group, and the CIBERSORT analysis showed that BC samples in the DYNLT1 high group had a higher infiltration ratio of CD8+ T cells and follicular helper T cells than those in the DYNLT1 low group (Figure 6H).

### 3.6. DYNLT1 knockdown suppressed the colony-forming, proliferation, migratory, and invasion abilities of BC cells

**Figure 7:**
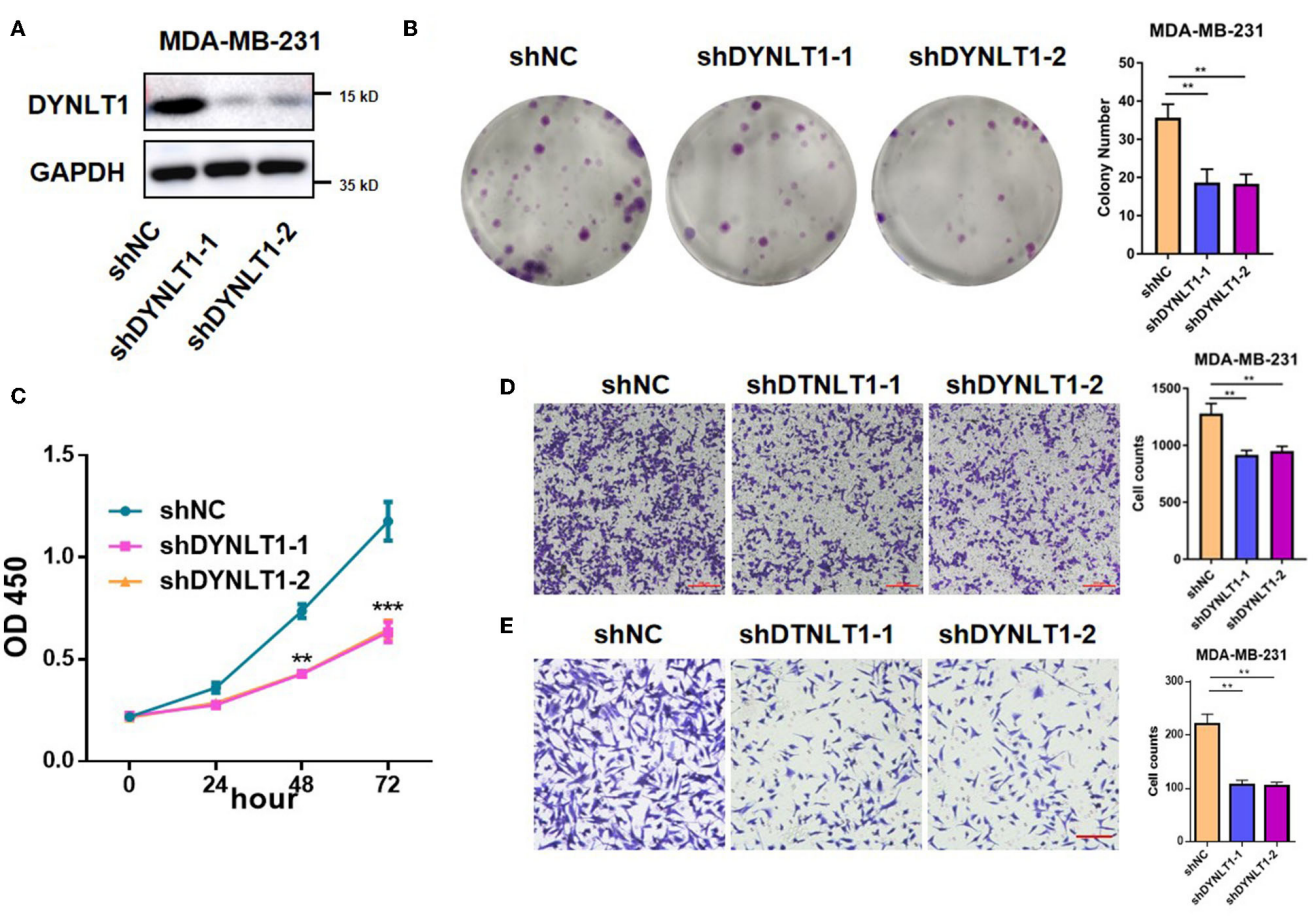
DYNLT1 knockdown inhibited MDA-MB-231 cells' colony formation and migration. **(A)** Knockdown expression of DYNLT1 in MDA-MB-231 cells. **(B)** DYNLT1 knockdown inhibited cell colony formation and histogram of colony numbers between the DYNLT1 knockdown group and control group. **(C)** CCK-8 assay of the DYNLT1 knockdown group and control group. **(D)** DYNLT1 knockdown inhibited cell migration and histogram of cell counts between the DYNLT1 knockdown group and control group. **(E)** DYNLT1 knockdown inhibited cell invasion and histogram of cell counts between the DYNLT1 knockdown group and the control group. Scale bar = 100 μm, ***P < 0.001, **P < 0.01.

To explore the functional role of DYNLT1 in BC cells, *in vitro* experiments were conducted. First, MDA-MB-231-shDYNLT1 and MDA-MB-231-sh scramble (NC) stable cell lines were constructed successfully by lentiviral transduction (Figure 7A). Next, the results of the colony-forming assay and CCK-8 assays demonstrated that knockdown expression of DYNLT1 inhibited the colony formation and proliferation abilities in MDA-MB-231 cells (Figures 7B, C). Furthermore, a transwell assay was conducted to confirm that migration and invasion abilities were also attenuated with the DYNLT1 knockdown in MDA-MB-231 cells (Figures 7D, E).

### 3.7. Knockdown of DYNLT1 suppressed tumor growth and abolished the lung and liver metastasis *in vivo*

**Figure 8:**
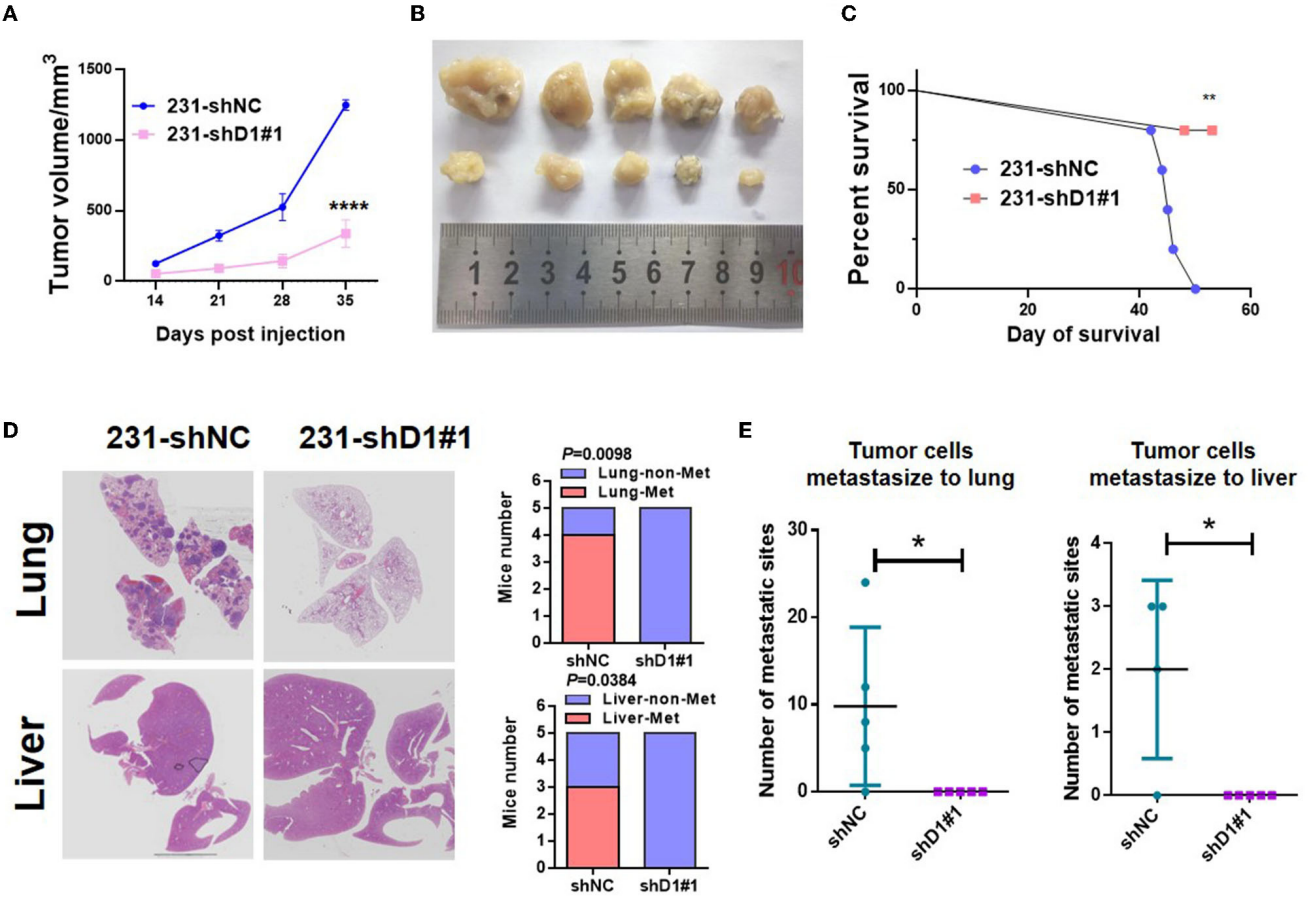
DYNLT1 knockdown suppressed tumor growth and abolished lung and liver metastasis *in vivo*. **(A)** DYNLT1 knockdown in MDA-MB-231 cells suppresses tumor growth in SCID mice. **(B)** Tumor samples were shown after mice were sacrificed. **(C)** Survival of SCID mice with MDA-MB-231-shNC tumor or MDA-MB-231-shDYNLT1#1 tumor. **(D)** Representative lung and liver HE staining of mice with MDA-MB-231-shNC tumor or MDA-MB-231-shDYNLT1#1 tumor (scale bar = 5 mm). **(E)** The number of tumor metastatic sites in the lung and the liver (*n* = 5 biological replicates). *P < 0.05, **P < 0.01, and ****P < 0.0001.

To determine the role of DYNLT1 *in vivo*, MDA-MB-231-shDYNLT1 and MDA-MB-231-shNC cells were injected into a mammary fat pad in severe combined immunodeficiency (SCID) female mice. Knockdown of DYNLT1 led to smaller tumor volume (Figures 8A, B) and poor survival (Figure 8C). The lung and the liver samples of sacrificed mice were formalin-fixed and paraffin-embedded, and HE stain was conducted to evaluate the distant metastasis. As expected, the knockdown of DYNLT1 prevented tumor cells from metastasizing to distant organs including the lung and the liver, which prolong the survival period of tumor-burden mice (Figures 8D, E).

## 4. Discussion

With the development of modern genomic/transcriptomic technologies and increasing public cancer genomic programs, such as TCGA and GEO, numerous biomarkers were identified for improving our ability to diagnose and treat cancer or utilized as predictors of prognosis and response to therapies in cancer. For example, DYNLT1 serving as a prognostic indicator for GBM patients has been reported (12). However, there is no report on whether DYNLT1 may act as a biomarker of BC.

In our study, we validated that DYNLT1 expression was higher in BC than normal breast tissues by integrated bioinformatics analysis using data from multiple public cohorts and ourselves BC and adjacent normal breast specimens. In addition, we demonstrated that high DYNLT1 expression meant a poor prognosis in BC, and DYNLT1 knockdown suppressed MDA-MB-231 cell migration and colony formation. Furthermore, in *in vivo* experiment, we found that DYNLT1 knockdown suppressed tumor growth and abolished distant metastasis. Mice with DYNLT1 knockdown tumor cells survived a longer period. However, there is still a limitation in our study. The molecular mechanism of how high DYNLT1 expression enhances BC cells' proliferative and invasive abilities remains unclear.

Abnormal proliferative and invasive abilities are the leading causes of progression and poor prognosis in BC. It has been reported that DYNLT1 promoted migration, invasion, and proliferation, and inhibited apoptosis of GC *via* the exo-miR-15b-3p/DYNLT1/Caspase-3/Caspase-9 pathway (13). Previous studies also found that DYNLT1 interacted with the tumor suppressor REIC/Dkk-3 which induced malignant cell death *via* modification of the Wnt signaling pathway (33, 34). In addition, Kawasaki et al. (35) demonstrated that REIC/Dkk-3 overexpression could induce multidrug-resistant BC cell line MCF-7 apoptosis *via* downregulating P-glycoprotein. Hence, a high expression of DYNLT1 can promote the progression of BC *via* interacting with REIC/Dkk-3.

Recent evidence indicates that a high proportion of TMB and loads of neoantigens predicted a good response for ICB therapy in many types of cancer (36, 37), and patients with high TILs and low TGF-beta response in tumor samples always carry a better prognostic significance in ICB treatment (38, 39). Our results showed that biomarkers representing an effective response to ICB treatment were always accompanied by DYNLT1 high expression in BC samples. Therefore, we inferred that DYNLT1 may be a response biomarker in ICB therapy for BC.

## 5. Conclusion

Our study first suggested that DYNLT1 may serve as a biomarker for diagnosing and ICB treating BC or a predictor for predicting the prognosis of BC.

## Data availability statement

The raw data supporting the conclusions of this article will be made available by the authors, without undue reservation.

## Ethics statement

The studies involving human participants were reviewed and approved by Ethics Committee of Affiliated Hospital of Jining Medical University (approval number: 2021-08-C015). The patients/participants provided their written informed consent to participate in this study. The animal study was reviewed and approved by the Ethics Committee of Affiliated Hospital of Jining Medical University, JNNL-202-DW-02.

## Author contributions

SM, GJ, CJ, and BX performed the experiments and wrote the manuscript. LihZ, RZ, HD, XY, LinZ, XP, and HZ collected the BC sample and performed the experiments. LijZ, LW, and TZ supervised the project and provided funds for the whole project. All authors approved the final manuscript.

## Supplementary material

Supplementary Table 1 Clinical information of patients.
