# Corrigendum: Identification of DYNLT1 associated with proliferation, relapse, and metastasis in breast cancer

**DOI:** 10.3389/fmed.2024.1375203

**Published:** 2024-02-05

**Authors:** Sen Miao, Gaoda Ju, Chonghua Jiang, Bing Xue, Lihua Zhao, Rui Zhang, Han Diao, Xingzhou Yu, Linlin Zhang, Xiaozao Pan, Hua Zhang, Lijuan Zang, Lei Wang, Tianhao Zhou

**Affiliations:** ^1^Department of Pathology, Affiliated Hospital of Jining Medical University, Jining, China; ^2^Department of Medical Oncology, Key Laboratory of Carcinogenesis and Translational Research (Ministry of Education/Beijing), Peking University Cancer Hospital and Institute, Beijing, China; ^3^Department of Neurosurgery, Affiliated Haikou Hospital of Xiangya Medical College, Central South University, Haikou, China; ^4^Department of Pathology Center, Shanghai General Hospital, Shanghai Jiaotong University School of Medicine, Shanghai, China; ^5^Department of Breast Surgery, Affiliated Hospital of Jining Medical University, Jining, China; ^6^Department of Medical Oncology, Shanghai First People's Hospital, Shanghai Jiao Tong University School of Medicine, Shanghai, China

**Keywords:** DYNLT1, breast cancer, biomarker, prognosis, immune checkpoint blocking therapy

In the published article, there was an error in affiliation of Dr. Tianhao Zhou. Instead of “1 Department of Pathology, Affiliated Hospital of Jining Medical University, Jining, China; 5 Department of Breast Surgery, Affiliated Hospital of Jining Medical University, Jining, China; 6 Key Laboratory of Breast Cancer Prevention and Treatment of the Ministry of Education, Tianjin Medical University Cancer Institute and Hospital, National Clinical Research Center of Cancer, Tianjin, China; 7 Department of Medical Oncology, Shanghai First People's Hospital, Shanghai Jiao Tong University School of Medicine, Shanghai, China”, it should be “6 Department of Medical Oncology, Shanghai First People's Hospital, Shanghai Jiao Tong University School of Medicine, Shanghai, China”.

The authors apologize for this error and state that this does not change the scientific conclusions of the article in any way. The original article has been updated.

