# Supplementary material for: Identification of DYNLT1 associated with proliferation, relapse, and metastasis in breast cancer
Source: Front Med (Lausanne). 2023 Apr 4;10:1167676. doi: 10.3389/fmed.2023.1167676 (PMC10110886; doi:10.3389/fmed.2023.1167676)
Supplement: Supplementary Table 1 — Clinical information of patients. [file Data_Sheet_1.pdf]

Supplementary Material

Identification of DYNLT1 associated with proliferation, relapse and metastasis in breast cancer

Sen Miao1#, Gaoda Ju2#, ChongHua Jiang3#, Bing Xue1#, Lihua Zhao1, Rui Zhang1, Han Diao1, Xingzhou Yu1, Linlin Zhang1, Xiaozao Pan1, Hua zhang1, Lijuan zang4*, Lei Wang5*, Tianhao Zhou[6, 7]*

*** Correspondence:** Tianhao Zhou: wwwzhou0809@163.com;

Lei Wang: Wang198111lei@126.com; Lijuan Zang: Lou19941205@163.com;

1. Original data

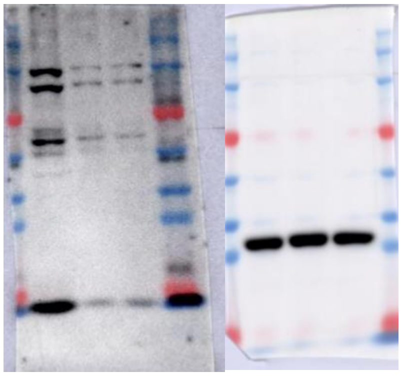

DYNLT, GAPDH

2 Supplementary Tables

Sample ID	Positive score	cell number score	IHC score	Tumor=1 , para-tumor=0, non-meligant=2	Relapse or metastasis relapse (0= no, 1= yes)	status(0=live, 1=death)	TNM Stage(0,I,II, III, IV)	pathologic grade	DFS	OS
1	1	2	2	1	0	0	IA	II	75	75
2	1	4	4	0	0	0	IIA	II	73	73
3	3	4	12	1	1	0	IIA	II	23	23

**4**	**1**	**1**	**1**	**0**	**0**	**1**	**IIA**	**II**	**9**	**9**
**5**	**1**	**3**	**3**	**1**	**0**	**1**	**IIA**	**II**	**78**	**78**
**6**	**2**	**4**	**8**	**0**	**0**	**1**	**IIA**	**II-III**	**20**	**20**
**7**	**3**	**4**	**12**	**1**	**0**	**1**	**IIA**	**II-III**	**20**	**20**
**8**	**2**	**3**	**6**	**0**	**1**	**1**	**IA**	**II**	**50**	**58**
**9**	**2**	**4**	**8**	**1**	**1**	**1**	**IA**	**II**	**50**	**58**
**10**	**2**	**3**	**6**	**1**	**1**	**0**	**IIA**	**I**	**59**	**59**
**11**	**2**	**4**	**8**	**0**	**1**	**0**	**IA**	**II-III**	**60**	**60**
**12**	**1**	**4**	**4**	**1**	**1**	**0**	**IA**	**II-III**	**60**	**60**
**13**	**1**	**3**	**3**	**0**	**1**	**0**	**IIIA**	**II**	**52**	**52**
**14**	**2**	**4**	**8**	**1**	**1**	**0**	**IIIA**	**II**	**52**	**52**
**15**	**2**	**3**	**6**	**1**	**1**	**0**	**IIA**	**II**	**50**	**50**
**16**	**2**	**4**	**8**	**0**	**0**	**0**	**IA**	**I**	**53**	**55**
**17**	**1**	**4**	**4**	**0**	**1**	**0**	**IA**	**II**	**21**	**48**
**18**	**3**	**4**	**12**	**1**	**1**	**0**	**IA**	**II**	**21**	**48**
**19**	**1**	**3**	**3**	**0**	**0**	**0**	**IIA**	**II**	**83**	**83**
**20**	**2**	**4**	**8**	**1**	**0**	**0**	**IIA**	**II**	**83**	**83**
**21**	**1**	**4**	**4**	**0**	**0**	**0**	**IIA**	**II**	**75**	**75**
**22**	**2**	**4**	**8**	**1**	**0**	**0**	**IIA**	**II**	**75**	**75**
**23**	**0**	**0**	**0**	**0**	**0**	**1**	**IIA**	**II-III**	**20**	**20**

24	1	3	3	1	0	1	IIA	II-III	20	20
25	1	1	1	0	0	0	IIA	II	73	73
26	1	4	4	1	0	0	IIA	II	73	73
27	2	3	6	0	0	0		无	73	73
28	2	4	8	1	0	0	IIA	无	73	73
29	1	4	4	0	0	0	IA	II	73	73
30	2	4	8	1	0	0	IA	II	73	73
31	1	4	4	0	0	0	IIA	II	74	74
32	2	4	8	1	0	0	IIA	II	74	74
33	1	3	3	0	0	0	IA	II	74	74
34	2	2	4	1	0	0	IA	II	74	74
35	1	4	4	0	0	0	IA	III	77	77
36	2	2	4	1	0	0	IA	III	77	77
37	0	0	0	0	0	0	I	无	69	69
38	1	4	4	1	0	0	IB	无	69	69
39	1	2	2	0	0	0	IIA	II	67	67
40	1	3	3	1	0	0	IIA	II	67	67
41	2	4	8	1	0	0	IIA	II	75	75
42	3	4	12	0	0	0	II	II	75	75
43	1	2	2	1	0	0	IA	II	72	72

**44**	**2**	**4**	**8**	**0**	**0**	**0**	**IA**	**II**	**72**	**72**
**45**	**1**	**4**	**4**	**1**	**0**	**0**	**IA**	**I**	**74**	**74**
**46**	**1**	**4**	**4**	**0**	**0**	**0**	**IA**	**I**	**74**	**74**
**47**	**0**	**0**	**0**	**0**	**0**	**0**	**IIIA**	**II-III**	**65**	**65**
**48**	**0**	**0**	**0**	**0**	**1**	**0**	**IIA**	**II-III**	**18**	**60**
**49**	**0**	**0**	**0**	**0**	**0**	**1**	**IIB**	**III**	**26**	**26**
**50**	**3**	**4**	**12**	**1**	**0**	**1**	**IIB**	**III**	**26**	**26**
**51**	**0**	**0**	**0**	**0**	**1**	**0**	**IA**	**II**	**31**	**48**
**52**	**0**	**0**	**0**	**0**	**1**	**0**	**IA**	**II**	**35**	**48**
**53**	**2**	**3**	**6**	**1**	**1**	**0**	**IA**	**II**	**55**	**48**
**54**	**0**	**0**	**0**	**0**	**0**	**0**	**IIIA**	无	**43**	**43**
**55**	**1**	**4**	**4**	**1**	**0**	**0**	**IIIA**	无	**43**	**43**
**56**	**1**	**4**	**4**	**0**	**0**	**0**	**IA**	**II**	**42**	**42**
**57**	**3**	**4**	**12**	**1**	**0**	**0**	**IA**	**II**	**42**	**42**
**58**	**2**	**4**	**8**	**0**	**0**	**0**	**IA**	**III**	**72**	**72**
**59**	**3**	**4**	**12**	**1**	**0**	**0**	**IA**	**III**	**72**	**72**
**60**	**1**	**4**	**4**	**0**	**0**	**0**	**IA**	**II**	**75**	**75**
**61**	**1.5**	**4**	**6**	**1**	**0**	**0**	**IA**	**II**	**75**	**75**
**62**	**1**	**4**	**4**	**0**	**0**	**0**	**I**	**II**	**75**	**75**
**63**	**2**	**4**	**8**	**1**	**0**	**0**	**IIA**	**II**	**75**	**75**

64	1	4	4	1	0	0	IA	无	73	73
65	1	1	1	0	0	0	IA	I	76	76
66	1	4	4	1	0	0	IA	I	76	76
67	1	2	2	0	0	0	IIA	无	82	82
68	1	4	4	1	0	0	IIA	无	82	82
69	1	4	4	0	0	0	IA	无	74	74
70	1	1	1	1	0	0	IA	无	74	74
71	1	3	3	0	0	0	IIIA	II	70	70
72	2	4	8	1	1	0	IIIA	II	43	43
73	1	4	4	0	0	0	IIA	III	78	78
74	1	4	4	1	0	0	IIA	III	78	78
75	1	4	4	1	0	0	IB	II	82	82
76	1	4	4	0	0	0		无	74	74
77	2	4	8	1	0	0	IIA	无	74	74
78	1	4	4	0	0	0	IIB	II	73	73
79	1	1	1	1	0	0	IB	II	73	73
80	2	4	8	1	0	0	IA	II	72	72
81	1	4	4	0	0	0	IA	II	70	70
82	2	4	8	1	0	0	IA	II	70	70
83	2	4	8	0	0	0	IIA	II	81	81

**84**	**3**	**4**	**12**	**1**	**0**	**0**	**IIA**	**II**	**81**	**81**
**85**	**0**	**0**	**0**	**0**	**0**	**0**	**IIA**	**II**	**80**	**80**
**86**	**2**	**4**	**8**	**1**	**0**	**0**	**IIA**	**II**	**80**	**80**
**87**	**2**	**4**	**8**	**0**	**0**	**0**	**IIA**	**III**	**78**	**78**
**88**	**2**	**4**	**8**	**1**	**0**	**0**	**IIA**	**III**	**78**	**78**
**89**	**2**	**2**	**4**	**0**	**0**	**0**	**IIA**	**Ⅲ**	**66**	**66**
**90**	**2**	**4**	**8**	**1**	**0**	**0**	**IIA**	**Ⅲ**	**66**	**66**
**91**	**1**	**4**	**4**	**0**	**0**	**0**	**IIA**	**Ⅱ**	**75**	**75**
**92**	**2**	**4**	**8**	**1**	**0**	**0**	**IIA**	**Ⅱ**	**75**	**75**
**93**	**1**	**4**	**4**	**1**	**0**	**0**	**IA**	**Ⅱ**	**69**	**69**
**94**	**1**	**2**	**2**	**0**	**0**	**0**	**I**	**Ⅱ**	**69**	**69**
**95**	**1**	**3**	**3**	**0**	**0**	**0**	**IA**	**Ⅱ**	**71**	**71**
**96**	**1**	**1**	**1**	**1**	**0**	**0**	**IA**	**Ⅱ**	**71**	**71**
**97**	**3**	**4**	**12**	**1**	**1**	**1**	**IIIA**	**II-III**	**33**	**33**
**98**	**1**	**4**	**4**	**1**	**1**	**0**	**IA**	**II**	**79**	**79**
**99**	**2**	**4**	**8**	**1**	**0**	**1**	**II**	**MISS**	**54**	**54**
**100**	**3**	**4**	**12**	**1**	**1**	**1**	**III**	**II**	**22**	**32**
**101**	**0**	**0**	**0**	**0**	**1**	**1**		**II**	**22**	**32**
**102**	**2.5**	**4**	**10**	**1**	**1**	**1**	**IIB**	**II**	**35**	**35**

103	3	4	12	1	1	0	IIB	II	42	42
104	1	4	4	1	0	1	II	II-III	43	43
105	3	4	12	1	1	1	IIIA	I-II	36	36
106	2	2	4	1	0	1	IA	II-III	77	77
107	3	4	12	1	0	0	IIA	III	72	72
108	3	4	12	1	1	1	IIA	MISS	25	25
109	1	4	4	1	0	1	IIB	II	78	78
110	2	4	8	1	0	1	IIB	III	19	19
111	2	3	6	1	0	1	IA	III	79	79
112	1	4	4	1	1	1	IA	III	43	43
113	1	1	1	0	1	1	IA	III	43	43
114	1	1	1	1	0	0	IIA	I	49	49
115	1	4	4	0		1	ⅢC	II	38	38
116	2	4	8	2	N/A	N/A	N/A	N/A	N/A	N/A
117	2	4	8	2	N/A	N/A	N/A	N/A	N/A	N/A
118	0	0	0	2	N/A	N/A	N/A	N/A	N/A	N/A
119	2	3	6	1	0	0	IB	II	35	35
120	1	4	4	2	N/A	N/A	N/A	N/A	N/A	N/A

**121**	**1**	**3**	**3**	**1**	**0**	**0**	**I**	**II**	**45**	**45**
**122**	**2**	**4**	**8**	**0**	**1**	**1**	**III**	**II**	**25**	**25**
**123**	**3**	**4**	**12**	**1**	**1**	**1**	**III**	**II**	**25**	**25**
